# Crystal structure and Hirshfeld surface analysis of bis­(benzoato-κ^2^ *O*,*O*′)[bis­(pyridin-2-yl-κ*N*)amine]nickel(II)

**DOI:** 10.1107/S2056989019010880

**Published:** 2019-08-13

**Authors:** Phichitra Phiokliang, Phakamat Promwit, Kittipong Chainok, Nanthawat Wannarit

**Affiliations:** a Department of Chemistry, Faculty of Science and Technology, Thammasat, University, Klong Luang, Pathum Thani 12121, Thailand; b Materials and Textile Technology, Faculty of Science and Technology, Thammasat University, Klong Luang, Pathum Thani 12121, Thailand

**Keywords:** nickel(II) complex, benzoate, bis­(pyridin-2-yl)amine, crystal structure, Hirshfeld surface analysis

## Abstract

A new mononuclear Ni^II^ complex with bis­(pyridin-2-yl)amine (dpyam) and benzoate (benz), [Ni(C_7_H_5_O_2_)_2_(C_10_H_9_N_3_)], crystallizes in the monoclinic space group *P*2_1_/*c*. The Ni^II^ ion adopts a *cis*-distorted octa­hedral geometry with an [NiN_2_O_4_] chromophore. In the crystal, the complex mol­ecules are linked together into a one-dimensional chain by symmetry-related π–π stacking inter­actions [centroid-to-centroid distance = 3.7257 (17) Å], along with N—H⋯O and C—H⋯O hydrogen bonds. The crystal packing is further stabilized by C—H⋯π inter­actions, which were investigated by Hirshfeld surface analysis.

## Abstract 2

The crystal structure of a new mononuclear Ni^II^ complex with bis­(pyridin-2-yl)amine (dpyam) and benzoate (benz) is reported.

## Chemical context   

Nickel(II) complexes have been of wide inter­est in many fields such as coordination chemistry (Devereux *et al.*, 2007 ▸; Lee *et al.*, 2012 ▸) and bioinorganic chemistry (Morgant *et al.*, 2006 ▸; Luo *et al.*, 2007 ▸; Zianna *et al.*, 2016 ▸), to name just a few. Generally, an Ni^II^ ion is stable in its [Ar]3*d* ^8^ electronic configuration. Among the various types of Ni^II^ complexes, mononuclear Ni^II^ complexes containing mixed carboxyl­ate and *N*-donor ligands have received considerable attention because of their inter­esting properties such as their behaviour catalysis in transesterification (Lee *et al.*, 2012 ▸) and their occasional bioactivity (Zianna *et al.*, 2016 ▸). One of the aims of our research group is to explore and study the coordination chemistry and bioactivities of new mononuclear complexes containing first row transition metal(II) ions and mixed ligands such as benzoate and *N*-donor bi­pyridine derivatives. Moreover we are interested in understanding the crystal structures and stability of the self-assembly between mononuclear units through non-covalent inter­actions, and the resulting properties of the material. Generally, a carboxyl­ate group of *e.g.* a benzoate can give rise to various types of coordination modes, leading to a variety of coordination geometries and coordination frameworks, while a phenyl ring is able to provide π–π stacking inter­actions that can support crystal stability. For *N*-donor ligands, bi­pyridine derivatives can act as chelating agents to form mononuclear units as building blocks for constructing 1D, 2D and 3D supra­molecular frameworks through weak inter­actions such as hydrogen bonding, π–π stacking among others, depending on the exact nature of the ligand. As part of our ongoing research into the coordination chemistry and bioactivities of new discrete Ni^II^ complexes containing benzoate and chelating *N*-donor ligands, we have synthesized a new mononuclear Ni^II^ complex containing benzoate (benz) and bis­(pyridin-2-yl)amine (dpyam) mixed ligands, [Ni(dpyam)(benz)_2_]. Herein, the crystal structure determination and Hirshfeld surface analysis of the title complex is reported.
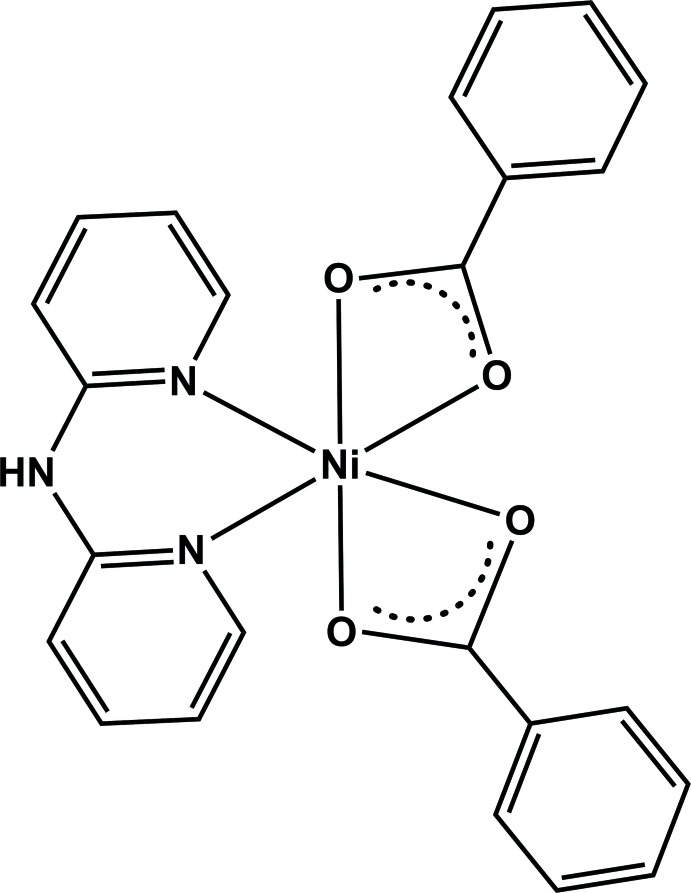


## Structural commentary   

**Figure 1:**
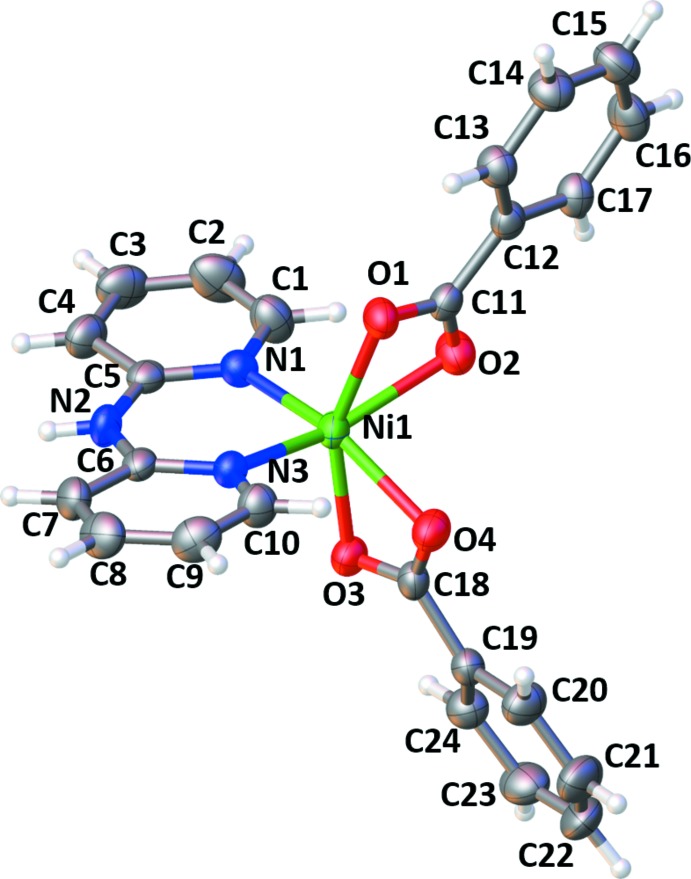
*ORTEP* representation of the title complex with the atom numbering. Displacement ellipsoids are drawn at the 50% probability level.

The title complex crystallizes in the monoclinic crystal system in the *P*2_1_/c space group. The asymmetric unit consists of one Ni^II^ ion, one dpyam, and two benzoate ligands. The Ni^II^ ion is six-coordinated by two nitro­gen atoms from the dpyam chelating ligand and four oxygen atoms from two benzoate chelating ligands, adopting a *cis*-distorted octa­hedral geometry as shown in Fig. 1 ▸. The Ni—N and Ni—O bond lengths range from 2.032 (2) to 2.045 (2) Å and 2.041 (2) to 2.221 (2) Å, respectively, whereas the bond angles around the central Ni atom are 61.53 (7)–159.84 (8)° (see Table 1 ▸). These values in the title complex are comparable to those of related Ni^II^ complexes such as [Ni(bpy)(benz)_2_] (bpy = 2,2′-bi­pyridine; Baruah *et al.*, 2007 ▸), and are shorter than those of other isostructural metal(II) complexes with the same ligand set, such as [*M*(dpyam)(benz)_2_], where *M* = Zn (Lee *et al.*, 2007 ▸), Cd (Park *et al.*, 2010 ▸) and Hg (Lee *et al.*, 2012 ▸), because of the different sizes of the central metal ions.

## Supra­molecular features   

**Figure 2:**
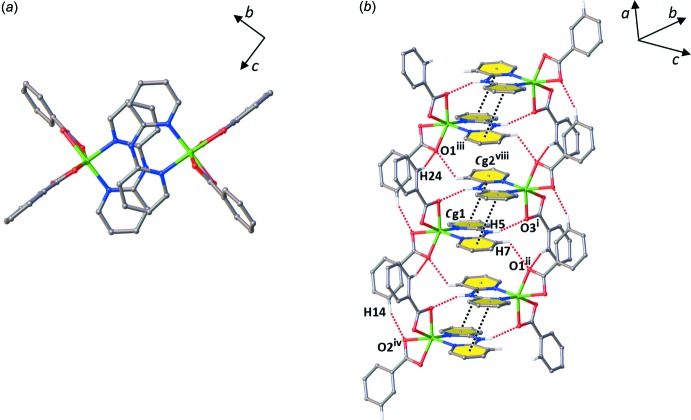
Views of lattice arrangement of the title complex along the [100] direction, (*a*) top view and (*b*) the weak inter­molecular inter­actions, N—H⋯O, C—H⋯O and π–π, between dimeric units, showing the one-dimensional supra­molecular chain-like structure.

**Figure 3:**
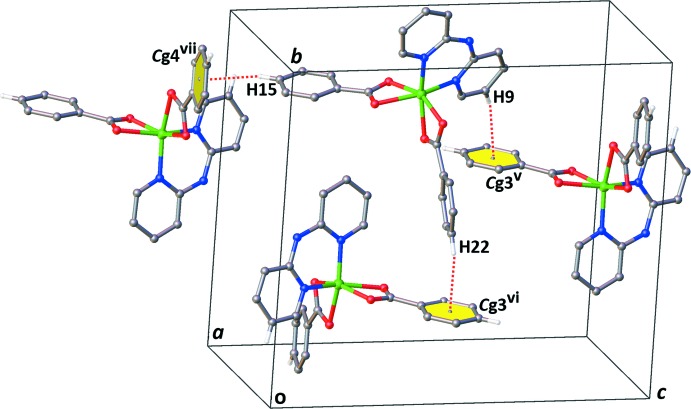
View of the C—H⋯π inter­molecular inter­actions of the title complex.

**Figure 4:**
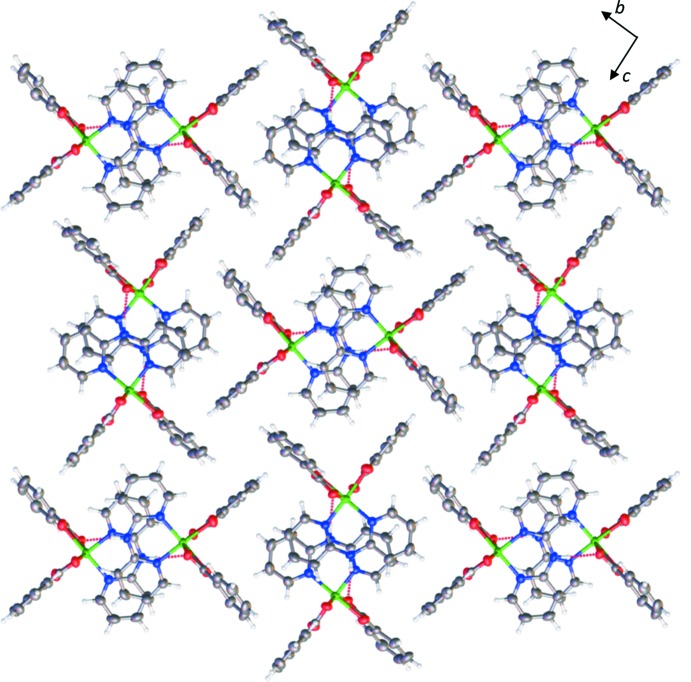
View of the three-dimensional supra­molecular network of the title complex.

In the crystal, adjacent complex mol­ecules are linked into dimeric species, Fig. 2 ▸ *a*, through aromatic π–π stacking inter­actions involving the pyridyl rings of the dpyam ligands with a centroid-to-centroid distance of 3.7257 (17) Å [*Cg*1⋯*Cg*2^viii^, symmetry code: (viii) −*x*, 2 − *y*, 1 − *z*; *Cg*1 and *Cg*2 are the centroids of the N1/C1–C5 and N3/C6–C10 rings, respectively] and an inter­planar spacing between dpyam ligands of 3.448 (2) Å. The π-stacking inter­action is augmented by a pair of inversion-symmetry-equivalent N—H⋯O hydrogen bonds (Table 2 ▸) between the NH and carboxyl­ate groups. The dimers are linked into a chain along the *a* axis *via* C—H⋯O hydrogen-bonding inter­actions, C—H_(dpyam)_⋯O1_(benzoate)_ and C—H_(benzoate)_⋯O_(benzoate)_, as shown in Fig. 2 ▸ *b*. C—H⋯O inter­actions are augmented by a second set of weaker π–π inter­actions that alternate with the first along the chain direction, Fig. 2 ▸ *b*. The latter set of weak π-stacking inter­actions presents a centroid-to-centroid distance of 4.3565 (17) Å [*Cg*1⋯*C*g2^ix^, symmetry code: (ix) 1 − *x*, 2 − *y*,1 − *z*] and an inter­planar spacing between dpyam ligands of 3.492 (3) Å. The chains are further connected by C—H⋯π inter­actions in the *bc* plane, Fig. 3 ▸, giving rise to a three-dimensional supra­molecular network, Fig. 4 ▸.

## Hirshfeld surface analysis   

**Figure 5:**
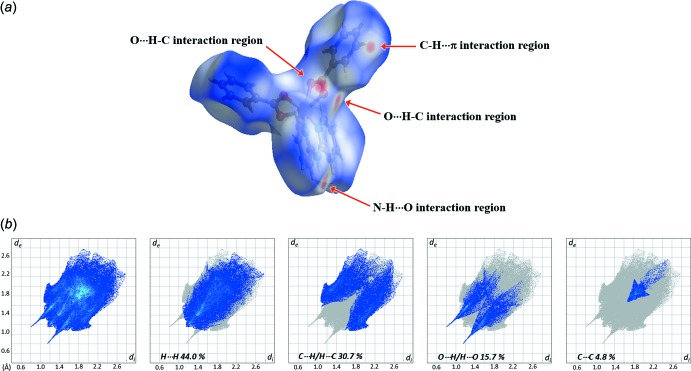
Views of (*a*) the Hirshfeld surface mapped over *d*_norm_ in the range −0.526 to +1.5208 (arbitrary units) and (*b*) Hirshfeld surface fingerprint plots for the H⋯H, C⋯H/H⋯C, O⋯H/H⋯O and C⋯C contacts of the title complex.

The inter­actions stabilizing the supra­molecular framework of the title complex have been further studied by the analysis of the Hirshfeld surfaces and their two-dimensional fingerprint plots. These results were visualized using the program *CrystalExplorer* (Turner *et al.*, 2017 ▸). The three-dimensional Hirshfeld surface of the title complex is shown in Fig. 5 ▸ *a*. Inter­actions are represented using different colours, red indicating distances closer than the sum of the van der Waals radii, white indicating distances near the van der Waals radii separation, and blue indicating distances longer than the van der Waals radii (McKinnon *et al.*, 2007 ▸; Venkatesan *et al.*, 2016 ▸). The strong inter­molecular N—H⋯O and C—H⋯O hydrogen bonding and C—H⋯π inter­actions in the crystal of the title complex are represented as red spots on *d*_norm_. Selected two-dimensional fingerprint plots are shown in Fig. 5 ▸ *b* for all contacts as well as individual H⋯H, C⋯H/H⋯C, O⋯H/H⋯O and C⋯C contacts, whose percentage contribution is also given. H⋯H inter­molecular contacts make the highest percentage contribution (44.0%), a result of the prevalence of hydrogen from the organic ligand. The C⋯H/H⋯C and O⋯H/H⋯O inter­molecular contacts are due to the attractive C—H⋯π and hydrogen-bonding inter­actions with percentage contrib­utions of 30.7 and 15.7%, respectively, indicating these to be the dominant stabilizing inter­actions in this crystal. The C⋯C contacts, with a percentage contribution of only 4.8%, indicate that the π–π inter­actions in the crystal of the title complex are weak compared to the other types of inter­actions, despite their prominent apparent role when visually inspecting the crystal structure.

## Characterization   

The IR spectrum (see Fig. S1 in the supporting information) of the title complex presents characteristic peaks at 3323, 3219 and 3148 cm^−1^ for N—H stretching and 1642 cm^−1^ for N—H bending, 1595 cm^−1^ for C=N aromatic stretching and 1421 cm^−1^ for C—N stretching in the coordinated dpyam ligand. Asymmetric and symmetric COO^−^ peaks of the chelating benzoate ligand are present at 1528 and 1489 cm^−1^, respectively. The peaks at 865, 772 and 687 cm^−1^ are assigned to C—H bending of aromatic rings. The peaks at 526 and 443 cm^−1^ have been assigned to Ni—O and Ni—N stretching, respectively (Zianna *et al.*, 2016 ▸).

The solid-state diffuse reflectance spectrum (Fig. S2) of the title complex presents three peaks at 391, 669 and 1044 nm that can be attributed to the allowed transitions ^3^ *A*_2g_ → ^3^ *T*_1g_(P), ^3^ *A*_2g_ → ^3^ *T*_1g_(F) and ^3^ *A*_2g_ → ^3^ *T*_2g_, respectively. In addition, the spectrum also shows a shoulder peak at 793 nm which can be attributed to a forbidden transition, ^3^ *A*_2g_ → ^1^ *E*_g_. This spectroscopic feature agrees with the typical *d*–*d* transitions of the Ni^II^ ion in a distorted octa­hedral geometry (Al-Riyahee *et al.*, 2018 ▸).

A PXRD pattern of the title complex was collected at room temperature (Fig. S3). The result shows that the pattern of the as-synthesized bulk material matches its simulated pattern, confirming the phase purity of the title complex.

## Database survey   

Previously reported complexes related to the title complex are [*M*(dpyam)(benz)_2_], *M* = Zn [CSD (Groom *et al.*, 2016 ▸) refcode GIJMAO; Lee *et al.*, 2007 ▸], Cd (WUVGOK; Park *et al.*, 2010 ▸) and Hg (QATXUG; Lee *et al.*, 2012 ▸). These complexes are isostructural. However, the size of the metal center in these complexes affects the metal-to-ligand distances (Alvarez, 2015 ▸) with the *M*—O/N bond lengths following the order Ni^II^ < Zn^II^ < Cd^II^ < Hg^II^ in the corresponding complexes, leading to a different degree of distortion in their coordination spheres.

## Synthesis and crystallization   

A methano­lic solution (15 mL) of dpyam (0.1712 g, 1 mmol) was slowly added into a warmed solution of Ni(NO_3_)_2_·6H_2_O (0.2908 g, 1 mmol) in distilled water (5 mL), under constant stirring for about 15 min; the resulting solution was kept at 333 K. Subsequently, solid sodium benzoate (0.2882 g, 2 mmol) was added slowly, resulting in a green precipitate. Then DMF (15 mL) was added dropwise and the solution was stirred until it became clear and green in colour. The solution mixture was filtered and left to stand at room temperature in air for slow evaporation. After a day, light-green rod-shaped crystals were obtained, collected by filtration, and air-dried [30.3% yield based on nickel(II) salt]. Elemental analysis calculated for C_24_H_19_NiN_3_O_4_: C, 60.80; H, 4.46; N, 8.86. Found: C, 60.56; H, 5.01; N, 8.26. IR (KBr, ν/cm^−1^): 3323*w*, 3219*w*, 3148*w*, 1642*m*, 1595*m*, 1541*s*, 1528*s*, 1489*s*, 1421*s*, 1242*w*, 1158*w*, 2021*w*, 865*w*, 772*m*, 729*m*, 687*w*, 526*w*, 443*w*.

## Refinement   

Crystal data, data collection and structure refinement details are summarized in Table 3 ▸. All hydrogen atoms were generated geometrically and refined isotropically using a riding model, with C—H = 0.93 Å and *U*_iso_(H) = 1.2*U*_eq_(C). The H atom bonded to the N atom of dpyam was located in a difference-Fourier map and was freely refined.

## Supplementary Material

Crystal structure: contains datablock(s) I. DOI: 10.1107/S2056989019010880/zl2759sup1.cif

Structure factors: contains datablock(s) I. DOI: 10.1107/S2056989019010880/zl2759Isup2.hkl

Supplementary figures. DOI: 10.1107/S2056989019010880/zl2759sup3.docx

CCDC reference: 1945125

Additional supporting information:  crystallographic information; 3D view; checkCIF report

## Figures and Tables

**Table 1:** Selected geometric parameters (Å, °)

Ni1—O1	2.0414 (18)	Ni1—O4	2.1540 (19)
Ni1—O2	2.2208 (19)	Ni1—N1	2.045 (2)
Ni1—O3	2.1050 (18)	Ni1—N3	2.032 (2)
			
O1—Ni1—O2	61.53 (7)	N1—Ni1—O3	98.10 (8)
O1—Ni1—O3	154.01 (7)	N1—Ni1—O4	159.84 (8)
O1—Ni1—O4	97.18 (7)	N3—Ni1—O1	97.87 (8)
O1—Ni1—N1	101.45 (8)	N3—Ni1—O2	159.25 (8)
O3—Ni1—O2	99.93 (7)	N3—Ni1—O3	98.67 (8)
O3—Ni1—O4	61.85 (7)	N3—Ni1—O4	93.84 (8)
O4—Ni1—O2	86.91 (7)	N3—Ni1—N1	91.21 (8)
N1—Ni1—O2	95.19 (8)		

**Table 2:** Hydrogen-bond geometry (Å, °) *Cg*3 and *Cg*4 are the centroids of the C12-C17 and C19–C24 rings, respectively.

*D*—H⋯*A*	*D*—H	H⋯*A*	*D*⋯*A*	*D*—H⋯*A*
C1—H1⋯O2	0.93	2.41	3.077 (4)	129
C10—H10⋯O4	0.93	2.49	3.038 (3)	118
N2—H5⋯O3^i^	0.83 (3)	2.12 (3)	2.913 (3)	159 (3)
C7—H7⋯O1^ii^	0.93	2.43	3.029 (3)	123
C24—H24⋯O1^iii^	0.93	2.44	3.338 (3)	162
C14—H14⋯O2^iv^	0.93	2.53	3.391 (3)	154
C9—H9⋯*Cg*3^v^	0.93	2.79	3.593 (3)	145
C22—H22⋯*Cg*3^vi^	0.93	2.89	3.671 (4)	143
C15—H15⋯*Cg*4^vii^	0.93	2.76	3.634 (3)	157

Symmetry codes: (i) 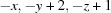; (ii) 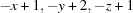; (iii) 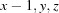; (iv) 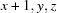; (v) 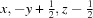; (vi) 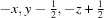; (vii) 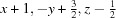.

**Table 3:** Experimental details

Crystal data	
Chemical formula	[Ni(C_7_H_5_O_2_)_2_(C_10_H_9_N_3_)]
*M*_r_	472.13
Crystal system, space group	Monoclinic, *P*2_1_/*c*
Temperature (K)	296
*a*, *b*, *c* (Å)	7.4199 (4), 16.679 (1), 17.6971 (11)
β (°)	100.778 (2)
*V* (Å^3^)	2151.5 (2)
*Z*	4
Radiation type	Mo *K*α
μ (mm^−1^)	0.94
Crystal size (mm)	0.32 × 0.20 × 0.20

Data collection	
Diffractometer	Bruker D8 QUEST CMOS
Absorption correction	Multi-scan (*SADABS*; Krause *et al.*, 2015 ▸)
*T*_min_, *T*_max_	0.655, 0.745
No. of measured, independent and observed [*I* > 2σ(*I*)] reflections	41528, 4407, 3569
*R*_int_	0.059
(sin θ/λ)_max_ (Å^−1^)	0.626

Refinement	
*R*[*F* ^2^ > 2σ(*F* ^2^)], *wR*(*F* ^2^), *S*	0.039, 0.096, 1.10
No. of reflections	4407
No. of parameters	293
H-atom treatment	H atoms treated by a mixture of independent and constrained refinement
Δρ_max_, Δρ_min_ (e Å^−3^)	0.45, −0.26

Computer programs: *APEX3* and *SAINT* (Bruker, 2016 ▸), *SHELXT* (Sheldrick, 2015*a* ▸), *SHELXL* (Sheldrick, 2015*b* ▸) and *OLEX2* (Dolomanov *et al.*, 2009 ▸).

## supplementary crystallographic information

### Crystal data

**Table:**

[Ni(C_7_H_5_O_2_)_2_(C_10_H_9_N_3_)]	*F*(000) = 976
*M**_r_* = 472.13	*D*_x_ = 1.458 Mg m^−^^3^
Monoclinic, *P*2_1_/*c*	Mo *K*α radiation, λ = 0.71073 Å
*a* = 7.4199 (4) Å	Cell parameters from 9934 reflections
*b* = 16.679 (1) Å	θ = 3.1–26.4°
*c* = 17.6971 (11) Å	µ = 0.94 mm^−^^1^
β = 100.778 (2)°	*T* = 296 K
*V* = 2151.5 (2) Å^3^	Block, light green
*Z* = 4	0.32 × 0.20 × 0.20 mm

### Data collection

**Table:**

Bruker D8 QUEST CMOS diffractometer	4407 independent reflections
Radiation source: sealed x-ray tube, Mo	3569 reflections with *I* > 2σ(*I*)
Graphite monochromator	*R*_int_ = 0.059
Detector resolution: 7.39 pixels mm^-1^	θ_max_ = 26.4°, θ_min_ = 3.1°
ω and φ scans	*h* = −9→9
Absorption correction: multi-scan (SADABS; Krause *et al.*, 2015)	*k* = −20→20
*T*_min_ = 0.655, *T*_max_ = 0.745	*l* = −21→22
41528 measured reflections	

### Refinement

**Table:**

Refinement on *F*^2^	0 restraints
Least-squares matrix: full	Hydrogen site location: mixed
*R*[*F*^2^ > 2σ(*F*^2^)] = 0.039	H atoms treated by a mixture of independent and constrained refinement
*wR*(*F*^2^) = 0.096	*w* = 1/[σ^2^(*F*_o_^2^) + (0.0327*P*)^2^ + 2.1013*P*] where *P* = (*F*_o_^2^ + 2*F*_c_^2^)/3
*S* = 1.10	(Δ/σ)_max_ = 0.001
4407 reflections	Δρ_max_ = 0.45 e Å^−^^3^
293 parameters	Δρ_min_ = −0.26 e Å^−^^3^

### Special details

**Table:**

Geometry. All esds (except the esd in the dihedral angle between two l.s. planes) are estimated using the full covariance matrix. The cell esds are taken into account individually in the estimation of esds in distances, angles and torsion angles; correlations between esds in cell parameters are only used when they are defined by crystal symmetry. An approximate (isotropic) treatment of cell esds is used for estimating esds involving l.s. planes.

### Fractional atomic coordinates and isotropic or equivalent isotropic displacement parameters (Å^2^)

**Table:**

	*x*	*y*	*z*	*U*_iso_*/*U*_eq_	
Ni1	0.16089 (4)	0.89490 (2)	0.37031 (2)	0.03029 (11)	
O1	0.3922 (2)	0.89019 (11)	0.32357 (10)	0.0365 (4)	
O2	0.1306 (2)	0.87442 (12)	0.24456 (11)	0.0413 (5)	
O3	−0.0955 (2)	0.85283 (10)	0.38795 (11)	0.0361 (4)	
O4	0.1251 (2)	0.76753 (11)	0.38110 (11)	0.0399 (5)	
N1	0.0989 (3)	1.01439 (13)	0.36105 (12)	0.0342 (5)	
N2	0.1916 (3)	1.04290 (13)	0.49359 (14)	0.0370 (5)	
H5	0.192 (4)	1.0771 (18)	0.5276 (17)	0.038 (8)*	
N3	0.2849 (3)	0.90878 (12)	0.48206 (12)	0.0311 (5)	
C1	0.0251 (4)	1.04335 (18)	0.29092 (17)	0.0476 (7)	
H1	0.011333	1.008330	0.249348	0.057*	
C2	−0.0307 (5)	1.1207 (2)	0.2771 (2)	0.0568 (9)	
H2	−0.081425	1.137499	0.227602	0.068*	
C3	−0.0107 (5)	1.17345 (19)	0.3377 (2)	0.0526 (8)	
H3	−0.047199	1.226609	0.329818	0.063*	
C4	0.0635 (4)	1.14671 (17)	0.40961 (19)	0.0451 (7)	
H4	0.077819	1.181450	0.451414	0.054*	
C5	0.1180 (3)	1.06633 (15)	0.41994 (16)	0.0331 (6)	
C6	0.2787 (3)	0.97412 (15)	0.52449 (15)	0.0308 (6)	
C7	0.3584 (4)	0.97654 (17)	0.60236 (16)	0.0401 (6)	
H7	0.348812	1.022417	0.631221	0.048*	
C8	0.4500 (4)	0.91137 (19)	0.63562 (17)	0.0476 (7)	
H8	0.504579	0.912522	0.687384	0.057*	
C9	0.4619 (4)	0.84332 (18)	0.59242 (18)	0.0464 (7)	
H9	0.524297	0.798089	0.614168	0.056*	
C10	0.3787 (4)	0.84458 (17)	0.51625 (16)	0.0398 (6)	
H10	0.387062	0.799141	0.486652	0.048*	
C11	0.3028 (4)	0.87810 (14)	0.25627 (15)	0.0312 (6)	
C12	0.4056 (4)	0.87111 (15)	0.19153 (15)	0.0324 (6)	
C13	0.5960 (4)	0.87371 (17)	0.20625 (16)	0.0387 (6)	
H13	0.659395	0.877598	0.256682	0.046*	
C14	0.6917 (4)	0.87057 (19)	0.14667 (18)	0.0462 (7)	
H14	0.819301	0.871863	0.156973	0.055*	
C15	0.5983 (4)	0.8655 (2)	0.07180 (18)	0.0501 (8)	
H15	0.662834	0.864220	0.031531	0.060*	
C16	0.4094 (4)	0.86233 (19)	0.05656 (16)	0.0472 (7)	
H16	0.346645	0.858820	0.006008	0.057*	
C17	0.3128 (4)	0.86435 (16)	0.11630 (15)	0.0368 (6)	
H17	0.185439	0.861160	0.105890	0.044*	
C18	−0.0389 (4)	0.78057 (15)	0.38557 (14)	0.0319 (6)	
C19	−0.1686 (4)	0.71259 (16)	0.38617 (15)	0.0336 (6)	
C20	−0.1020 (4)	0.63507 (17)	0.39881 (19)	0.0477 (7)	
H20	0.023745	0.626021	0.410818	0.057*	
C21	−0.2220 (5)	0.5716 (2)	0.3936 (2)	0.0617 (10)	
H21	−0.176871	0.519815	0.402797	0.074*	
C22	−0.4075 (5)	0.5841 (2)	0.3750 (2)	0.0601 (9)	
H22	−0.487872	0.540837	0.369942	0.072*	
C23	−0.4746 (4)	0.6610 (2)	0.36369 (19)	0.0533 (8)	
H23	−0.600533	0.669629	0.352004	0.064*	
C24	−0.3556 (4)	0.72552 (18)	0.36964 (16)	0.0410 (6)	
H24	−0.401431	0.777426	0.362537	0.049*	

### Atomic displacement parameters (Å^2^)

**Table:**

	*U*^11^	*U*^22^	*U*^33^	*U*^12^	*U*^13^	*U*^23^
Ni1	0.03251 (18)	0.02790 (18)	0.03103 (18)	−0.00190 (14)	0.00746 (13)	−0.00557 (14)
O1	0.0347 (9)	0.0410 (10)	0.0339 (10)	−0.0049 (8)	0.0068 (8)	−0.0094 (8)
O2	0.0337 (10)	0.0496 (12)	0.0410 (11)	−0.0025 (9)	0.0077 (8)	−0.0100 (9)
O3	0.0393 (10)	0.0282 (10)	0.0421 (11)	−0.0021 (8)	0.0110 (8)	−0.0048 (8)
O4	0.0363 (11)	0.0343 (10)	0.0508 (12)	−0.0029 (8)	0.0123 (9)	−0.0086 (9)
N1	0.0378 (12)	0.0321 (12)	0.0342 (12)	0.0002 (9)	0.0105 (10)	−0.0002 (9)
N2	0.0498 (14)	0.0246 (11)	0.0371 (13)	−0.0012 (10)	0.0094 (11)	−0.0071 (10)
N3	0.0348 (11)	0.0265 (11)	0.0326 (11)	−0.0013 (9)	0.0081 (9)	−0.0023 (9)
C1	0.0588 (19)	0.0454 (17)	0.0389 (16)	0.0107 (15)	0.0104 (14)	0.0017 (13)
C2	0.067 (2)	0.055 (2)	0.0496 (19)	0.0178 (17)	0.0143 (16)	0.0191 (16)
C3	0.058 (2)	0.0359 (16)	0.067 (2)	0.0091 (14)	0.0201 (17)	0.0133 (15)
C4	0.0511 (17)	0.0294 (15)	0.0569 (19)	0.0016 (13)	0.0159 (15)	−0.0019 (13)
C5	0.0308 (13)	0.0308 (13)	0.0406 (15)	−0.0032 (11)	0.0144 (12)	−0.0003 (11)
C6	0.0289 (13)	0.0295 (13)	0.0358 (14)	−0.0071 (10)	0.0107 (11)	−0.0047 (11)
C7	0.0455 (16)	0.0397 (16)	0.0344 (14)	−0.0082 (13)	0.0054 (12)	−0.0094 (12)
C8	0.0427 (17)	0.059 (2)	0.0371 (16)	−0.0103 (14)	−0.0035 (13)	−0.0024 (14)
C9	0.0418 (16)	0.0445 (17)	0.0497 (18)	0.0020 (13)	0.0001 (14)	0.0087 (14)
C10	0.0434 (16)	0.0343 (15)	0.0411 (16)	0.0037 (12)	0.0068 (13)	−0.0015 (12)
C11	0.0349 (14)	0.0243 (13)	0.0340 (14)	−0.0024 (10)	0.0050 (11)	−0.0030 (10)
C12	0.0373 (14)	0.0260 (13)	0.0340 (14)	0.0014 (11)	0.0068 (11)	−0.0023 (10)
C13	0.0390 (15)	0.0419 (16)	0.0347 (14)	−0.0002 (12)	0.0052 (12)	−0.0025 (12)
C14	0.0369 (15)	0.0532 (18)	0.0503 (18)	0.0014 (13)	0.0125 (14)	−0.0025 (14)
C15	0.059 (2)	0.0552 (19)	0.0414 (17)	0.0046 (15)	0.0220 (15)	0.0014 (14)
C16	0.059 (2)	0.0511 (18)	0.0290 (14)	0.0066 (15)	0.0026 (13)	−0.0004 (13)
C17	0.0384 (15)	0.0356 (14)	0.0344 (15)	0.0026 (12)	0.0021 (12)	−0.0036 (11)
C18	0.0390 (15)	0.0291 (13)	0.0277 (13)	−0.0017 (11)	0.0066 (11)	−0.0056 (10)
C19	0.0406 (15)	0.0308 (14)	0.0309 (13)	−0.0033 (11)	0.0108 (11)	−0.0054 (11)
C20	0.0503 (18)	0.0350 (15)	0.061 (2)	−0.0004 (13)	0.0180 (15)	−0.0017 (14)
C21	0.078 (3)	0.0318 (16)	0.082 (3)	−0.0071 (16)	0.033 (2)	−0.0078 (16)
C22	0.072 (2)	0.048 (2)	0.066 (2)	−0.0305 (17)	0.0260 (18)	−0.0172 (16)
C23	0.0443 (17)	0.063 (2)	0.0533 (19)	−0.0164 (16)	0.0112 (15)	−0.0032 (16)
C24	0.0412 (16)	0.0417 (16)	0.0406 (16)	−0.0027 (13)	0.0086 (13)	−0.0007 (13)

### Geometric parameters (Å, º)

**Table:**

Ni1—O1	2.0414 (18)	C8—C9	1.381 (4)
Ni1—O2	2.2208 (19)	C9—H9	0.9300
Ni1—O3	2.1050 (18)	C9—C10	1.374 (4)
Ni1—O4	2.1540 (19)	C10—H10	0.9300
Ni1—N1	2.045 (2)	C11—C12	1.495 (4)
Ni1—N3	2.032 (2)	C12—C13	1.388 (4)
O1—C11	1.266 (3)	C12—C17	1.384 (4)
O2—C11	1.257 (3)	C13—H13	0.9300
O3—C18	1.280 (3)	C13—C14	1.378 (4)
O4—C18	1.253 (3)	C14—H14	0.9300
N1—C1	1.349 (4)	C14—C15	1.378 (4)
N1—C5	1.342 (3)	C15—H15	0.9300
N2—H5	0.83 (3)	C15—C16	1.378 (4)
N2—C5	1.372 (4)	C16—H16	0.9300
N2—C6	1.379 (3)	C16—C17	1.384 (4)
N3—C6	1.329 (3)	C17—H17	0.9300
N3—C10	1.356 (3)	C18—C19	1.489 (4)
C1—H1	0.9300	C19—C20	1.387 (4)
C1—C2	1.363 (4)	C19—C24	1.380 (4)
C2—H2	0.9300	C20—H20	0.9300
C2—C3	1.373 (5)	C20—C21	1.375 (4)
C3—H3	0.9300	C21—H21	0.9300
C3—C4	1.363 (4)	C21—C22	1.370 (5)
C4—H4	0.9300	C22—H22	0.9300
C4—C5	1.402 (4)	C22—C23	1.376 (5)
C6—C7	1.395 (4)	C23—H23	0.9300
C7—H7	0.9300	C23—C24	1.383 (4)
C7—C8	1.357 (4)	C24—H24	0.9300
C8—H8	0.9300		
			
O1—Ni1—O2	61.53 (7)	C7—C8—C9	119.8 (3)
O1—Ni1—O3	154.01 (7)	C9—C8—H8	120.1
O1—Ni1—O4	97.18 (7)	C8—C9—H9	121.1
O1—Ni1—N1	101.45 (8)	C10—C9—C8	117.8 (3)
O3—Ni1—O2	99.93 (7)	C10—C9—H9	121.1
O3—Ni1—O4	61.85 (7)	N3—C10—C9	123.3 (3)
O4—Ni1—O2	86.91 (7)	N3—C10—H10	118.4
N1—Ni1—O2	95.19 (8)	C9—C10—H10	118.4
N1—Ni1—O3	98.10 (8)	O1—C11—C12	118.7 (2)
N1—Ni1—O4	159.84 (8)	O2—C11—O1	120.0 (2)
N3—Ni1—O1	97.87 (8)	O2—C11—C12	121.2 (2)
N3—Ni1—O2	159.25 (8)	C13—C12—C11	120.1 (2)
N3—Ni1—O3	98.67 (8)	C17—C12—C11	120.7 (2)
N3—Ni1—O4	93.84 (8)	C17—C12—C13	119.3 (2)
N3—Ni1—N1	91.21 (8)	C12—C13—H13	119.8
C11—O1—Ni1	93.15 (15)	C14—C13—C12	120.5 (3)
C11—O2—Ni1	85.30 (15)	C14—C13—H13	119.8
C18—O3—Ni1	89.87 (15)	C13—C14—H14	120.0
C18—O4—Ni1	88.37 (15)	C13—C14—C15	120.0 (3)
C1—N1—Ni1	117.99 (19)	C15—C14—H14	120.0
C5—N1—Ni1	125.26 (18)	C14—C15—H15	120.0
C5—N1—C1	116.7 (2)	C16—C15—C14	120.0 (3)
C5—N2—H5	116 (2)	C16—C15—H15	120.0
C5—N2—C6	133.4 (2)	C15—C16—H16	119.9
C6—N2—H5	110 (2)	C15—C16—C17	120.2 (3)
C6—N3—Ni1	125.88 (18)	C17—C16—H16	119.9
C6—N3—C10	117.7 (2)	C12—C17—H17	120.0
C10—N3—Ni1	116.45 (17)	C16—C17—C12	120.1 (3)
N1—C1—H1	117.9	C16—C17—H17	120.0
N1—C1—C2	124.1 (3)	O3—C18—C19	120.0 (2)
C2—C1—H1	117.9	O4—C18—O3	119.6 (2)
C1—C2—H2	120.6	O4—C18—C19	120.3 (2)
C1—C2—C3	118.8 (3)	C20—C19—C18	120.0 (3)
C3—C2—H2	120.6	C24—C19—C18	120.3 (2)
C2—C3—H3	120.5	C24—C19—C20	119.6 (3)
C4—C3—C2	119.0 (3)	C19—C20—H20	120.0
C4—C3—H3	120.5	C21—C20—C19	120.0 (3)
C3—C4—H4	120.3	C21—C20—H20	120.0
C3—C4—C5	119.4 (3)	C20—C21—H21	119.7
C5—C4—H4	120.3	C22—C21—C20	120.5 (3)
N1—C5—N2	121.3 (2)	C22—C21—H21	119.7
N1—C5—C4	122.0 (3)	C21—C22—H22	120.1
N2—C5—C4	116.7 (3)	C21—C22—C23	119.8 (3)
N2—C6—C7	116.6 (2)	C23—C22—H22	120.1
N3—C6—N2	121.4 (2)	C22—C23—H23	119.8
N3—C6—C7	121.9 (2)	C22—C23—C24	120.4 (3)
C6—C7—H7	120.3	C24—C23—H23	119.8
C8—C7—C6	119.4 (3)	C19—C24—C23	119.8 (3)
C8—C7—H7	120.3	C19—C24—H24	120.1
C7—C8—H8	120.1	C23—C24—H24	120.1
			
Ni1—O1—C11—O2	0.3 (2)	C3—C4—C5—N1	−0.2 (4)
Ni1—O1—C11—C12	178.36 (19)	C3—C4—C5—N2	−180.0 (3)
Ni1—O2—C11—O1	−0.3 (2)	C5—N1—C1—C2	−0.3 (4)
Ni1—O2—C11—C12	−178.3 (2)	C5—N2—C6—N3	−9.2 (4)
Ni1—O3—C18—O4	5.3 (2)	C5—N2—C6—C7	171.3 (3)
Ni1—O3—C18—C19	−173.2 (2)	C6—N2—C5—N1	9.4 (4)
Ni1—O4—C18—O3	−5.2 (2)	C6—N2—C5—C4	−170.8 (3)
Ni1—O4—C18—C19	173.3 (2)	C6—N3—C10—C9	2.0 (4)
Ni1—N1—C1—C2	176.8 (3)	C6—C7—C8—C9	−0.6 (4)
Ni1—N1—C5—N2	3.2 (3)	C7—C8—C9—C10	−0.1 (5)
Ni1—N1—C5—C4	−176.6 (2)	C8—C9—C10—N3	−0.6 (4)
Ni1—N3—C6—N2	−3.7 (3)	C10—N3—C6—N2	177.8 (2)
Ni1—N3—C6—C7	175.80 (19)	C10—N3—C6—C7	−2.7 (4)
Ni1—N3—C10—C9	−176.7 (2)	C11—C12—C13—C14	−177.4 (3)
O1—C11—C12—C13	3.3 (4)	C11—C12—C17—C16	176.5 (3)
O1—C11—C12—C17	−174.8 (2)	C12—C13—C14—C15	0.6 (5)
O2—C11—C12—C13	−178.7 (2)	C13—C12—C17—C16	−1.7 (4)
O2—C11—C12—C17	3.2 (4)	C13—C14—C15—C16	−1.0 (5)
O3—C18—C19—C20	−167.3 (3)	C14—C15—C16—C17	0.1 (5)
O3—C18—C19—C24	16.6 (4)	C15—C16—C17—C12	1.2 (4)
O4—C18—C19—C20	14.2 (4)	C17—C12—C13—C14	0.8 (4)
O4—C18—C19—C24	−161.9 (3)	C18—C19—C20—C21	−175.1 (3)
N1—C1—C2—C3	0.3 (5)	C18—C19—C24—C23	174.3 (3)
N2—C6—C7—C8	−178.4 (3)	C19—C20—C21—C22	0.9 (5)
N3—C6—C7—C8	2.1 (4)	C20—C19—C24—C23	−1.8 (4)
C1—N1—C5—N2	180.0 (2)	C20—C21—C22—C23	−2.0 (6)
C1—N1—C5—C4	0.2 (4)	C21—C22—C23—C24	1.2 (5)
C1—C2—C3—C4	−0.3 (5)	C22—C23—C24—C19	0.7 (5)
C2—C3—C4—C5	0.2 (5)	C24—C19—C20—C21	1.1 (5)

### Hydrogen-bond geometry (Å, º)

*Cg*3 and *Cg*4 are the centroids of the C12–C17 and C19-C24 rings, respectively.

**Table:**

*D*—H···*A*	*D*—H	H···*A*	*D*···*A*	*D*—H···*A*
C1—H1···O2	0.93	2.41	3.077 (4)	129
C10—H10···O4	0.93	2.49	3.038 (3)	118
N2—H5···O3^i^	0.83 (3)	2.12 (3)	2.913 (3)	159 (3)
C7—H7···O1^ii^	0.93	2.43	3.029 (3)	123
C24—H24···O1^iii^	0.93	2.44	3.338 (3)	162
C14—H14···O2^iv^	0.93	2.53	3.391 (3)	154
C9—H9···*Cg*3^v^	0.93	2.79	3.593 (3)	145
C22—H22···*Cg*3^vi^	0.93	2.89	3.671 (4)	143
C15—H15···*Cg*4^vii^	0.93	2.76	3.634 (3)	157

Symmetry codes: (i) −*x*, −*y*+2, −*z*+1; (ii) −*x*+1, −*y*+2, −*z*+1; (iii) *x*−1, *y*, *z*; (iv) *x*+1, *y*, *z*; (v) *x*, −*y*+1/2, *z*−1/2; (vi) −*x*, *y*−1/2, −*z*+1/2; (vii) *x*+1, −*y*+3/2, *z*−1/2.
