# Supplementary material for: Crystal structure and Hirshfeld surface analysis of bis­(benzoato-κ2 O,O′)[bis­(pyridin-2-yl-κN)amine]nickel(II)
Source: Acta Crystallogr E Crystallogr Commun. 2019 Aug 13;75(Pt 9):1301–5. doi: 10.1107/S2056989019010880 (PMC6727057; doi:10.1107/S2056989019010880)
Supplement: Supplementary file 3 [file e-75-01301-sup3.docx]

**Supporting Information**

**Crystal structure and Hirshfeld surface analysis of**

**bis(benzoato-κ^2^*O*,*O*′)(di(pyridin-2-yl)amine-κ^2^*N*,*N*′)nickel(II)**

Phichitra Phiokliang,^a^ Phakamat Promwit,^a^ Kittipong Chainok^b^ and Nanthawat Wannarit^a^*

**Figure Caption**

**

**

**Figure S1**

FT-IR spectrum of the title complex





**Figure S2**

Solid state diffuse reflectance spectrum of the title complex





**Figure S3**

Powder XRD patterns of the title complex


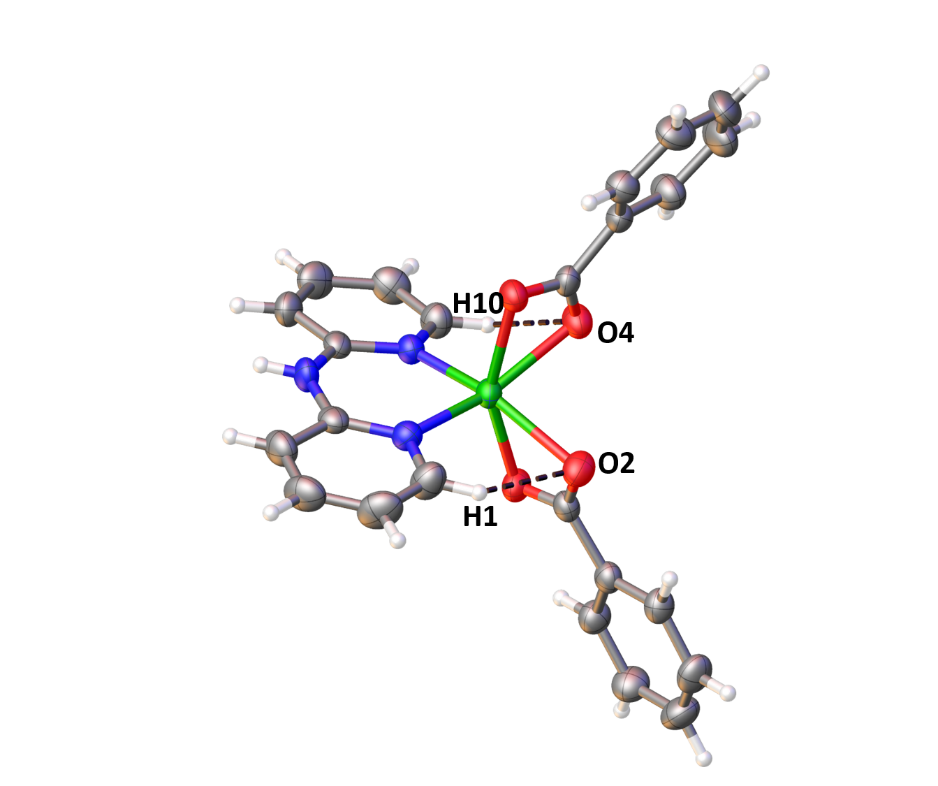


**Figure S4**

Intramolecular interactions of the title complex
